# Biochemical Activity and Hypoglycemic Effects of* Rumex obtusifolius* L. Seeds Used in Armenian Traditional Medicine

**DOI:** 10.1155/2018/4526352

**Published:** 2018-11-07

**Authors:** Anush Aghajanyan, Armine Nikoyan, Armen Trchounian

**Affiliations:** Department of Biochemistry, Microbiology and Biotechnology, Faculty of Biology, Yerevan State University, 0025 Yerevan, Armenia

## Abstract

Diabetes mellitus (DM) is a serious chronic metabolic disorder. Various diseases are being treated with medicinal plants and that is because of the less side effects of the current therapy. The diversity of plants in Armenia is due to the singularity of natural environment. However, biochemical activity of these plants has not been studied well. Thus, the goal was to investigate biochemical activity and antihyperglycemic properties of* Rumex obtusifolius *L. in rabbits with hyperglycemia. The high content of total phenolic compounds, flavonoids, and tannins has been determined in this plant extract. Oral administration of ethanol extract showed significant effect on hyperglycemia, reducing fasting glucose levels (57.3%, p<0.05), improving glucose tolerance, and increasing liver glycogen content (1.5-fold, p<0.01) compared to the hyperglycemic control group. Furthermore, ethanol extract of* R. obtusifolius* reduced total cholesterol, low-density lipoprotein cholesterol levels, and vice versa increased high-density lipoprotein cholesterol levels and also decreased liver enzymes levels (alanine aminotransferase and aspartate aminotransferase) compared with untreated group. These findings suggest that* R. obtusifolius* may have beneficial effects and should be supplement, as herbal remedy in the treatment of DM.

## 1. Introduction

Hyperglycemia is a metabolic disorder of the endocrine system characterized by abnormal glucose metabolism which is demonstrated by high blood sugar (hyperglycemia), hypercholesterolemia, high blood pressure, and cardiovascular disease due to the disturbances of carbohydrate, lipid, and protein metabolism resulting from insulin resistant and *β*-cell dysfunction [1–3]. Dyslipidemia is a very frequent metabolic disorder which is characterized by an increase of the rates of triglycerides (TG), total cholesterol (TC), low-density lipoprotein (LDL), and reduction of the high-density lipoprotein (HDL) [4–6]. It is known that hypercholesterolemia contributes to development of the atherosclerosis and subsequent of hypertension, ischemic heart disease, and renal failure [3, 6].

Immobilization stress can increase the blood glucose levels and the risk of cardiovascular diseases due to impaired endothelial function. Chronic immobilization stress leads to atherosclerosis, which has an endothelial dysfunction at early stages [7]. The oxidative damage and inflammatory mediators induced by chronic psychological stress play a key role in this process. Furthermore, durable immobilization can contribute to the formation of unstable atherosclerotic lesions as a result of immune system cells accumulation and molecules adhesion, leading to thrombosis and cardiac complications [7, 8].

There are many drugs currently used in the treatment of diabetes. Some reports indicate that treatments with synthetic antidiabetic drugs have toxicity and cause adverse effects, such as hypoglycemia, gastrointestinal and liver problems [9]. Secondary metabolites of plants are responsible for prevention and treatment of various diseases. Tissues of many plant species contain secondary metabolites such as flavonoids, glycosides, saponins, steroids, tannins, alkaloids, and terpenes. It is known that therapeutic effects of medicinal plants are specified due to biological active compounds. In fact, one part of the plant may exert a beneficial medicinal property, while other parts of the same plant may be ineffective or even toxic. Consequently, screening of different parts of plants with antihyperglycemic and antihyperlipidemic activities which may be effective in the treatment of diabetes mellitus would be of a great interest.

Armenian flora has large diversity of plants which might delay the development of diabetic complications and correct metabolic disorders. Thus, the identification of potent antidiabetic agents from natural sources, such as edible plants with minimum side effects for diabetic patients, is crucial. Plants belonging to the genus* Rumex* have been used traditionally either as edible plants or for the treatment of several diseases in many parts of the world [10].* Rumex *species are important edible and medicinal plants used in Armenian traditional medicine. These plant species have been used in folk medicine for the treatment of various diseases and ailments, including hypertension, inflammation, and bacterial infections [11, 12].* Rumex obtusifolius *(RO) has been shown to have antibacterial activity [11]. Methanol extract of the leaves showed cytotoxic and high antioxidant activity due to its phenolic content [13]. However biochemical properties and hypoglycemic effects of RO have been not studied thoroughly.

Thus, the goal was to investigate biochemical properties and antihyperglycemic properties of RO in rabbits with hyperglycemia.

## 2. Materials and Methods

### 2.1. Plant Material and Preparation of the Extract

The seeds of the plant were harvested after maturation. Identification of plant was carried out at the Department of Botany and Mycology, Yerevan State University, Yerevan (Armenia). A voucher specimen (No ERCB13208) has been deposited in the herbarium of Yerevan State University. The dried seeds were extracted with 40% ethanol for 20 min at 60°C. Briefly, 270-280 mg dry matter in 6 ml of ethanol. The extract was filtered with Watman filter paper (N1, Unichem, China) and after cooling at room temperature was orally administrated.

### 2.2. Quantitative Phytochemical Analysis

The total phenolic content (TPC) of crude extracts was measured using Folin-Ciocalteu method [14]. 2.5 mL 10% Folin-Ciocalteu reagent and 2.5 mL 7.5% Na_2_CO_3_ were added to 0.5 mL sample extract (200 mg/mL concentration). The reaction mixture was incubated at 45°C for 40 min, and afterwards absorption was measured at 765 nm wavelength (GENESYS 10S UV-VIS spectrophotometer, USA). Solvent was used, as blank. TPC of plant extracts was evaluated according to gallic acid calibration curve and expressed as micrograms of gallic acid equivalents per mg of dry extract (GAE/mg DW). Flavonoids were determined by the colorimetric method with AlCl_3_ [15]. 0.75 mL methanol, 0.05 mL 10% AlCl_3,_ 0.05 mL 1 M Na-K tartrate, and 1.4 mL distilled water were added to 0.25 mL plant extract (200 mg/mL concentration). The mixture was incubated at room temperature for 30 min; then absorption was measured at 415 nm wavelength, using quercetin, as standard. The amount of flavonoids expressed as micrograms of quercetin equivalent per mg of dry extract (QE/mg DW). For quantify of total tannins 3 mL of 4% vanillin (methanol solution) and 1.5 mL of concentrated HCl were added to 50 *μ*L plant extract (200 mg/ml) [16]. The mixture was incubated at room temperature for 15 min, and afterwards absorption was measured at 500 nm wavelength, methanol was used, as blank. Quantity of total tannins was expressed as micrograms of (+)-catechin equivalent per mg dry extract (CE/mg DW).

### 2.3. Animals

Nine domestic male rabbits (*Oryctolagus cuniculus domesticus*) (1800-1900 g) were used in this study, which was authorized by the “International Recommendation on Carrying out of Biomedical Researches with use of Animals,” and the study plan has been approved by the National Center of Bioethics (Armenia). They were acclimatized for 1 week prior to experimentation. The animals were housed under standard environmental conditions (temperature 22±2°C in a light/dark cycle of 12 h) and had free access to food and water during the experimental period. Initial body weights were recorded one day before the start of experiments.

### 2.4. Induction of Hyperglycemia in Experimental Rabbits and Blood Sampling

Hyperglycemia was induced by immobilization stress in the rabbits during 21 days (5 h daily) [7, 8]. They were roughly fixed on the board. Group 1 served as normoglycemic, group 2 was the hyperglycemic control (putting immobilization), and group 3— in common with immobilization—was administrated in single orally doses in 2 ml ethanol extract of RO. Blood glucose levels, lipid profile, and body weight of rabbits were measured at the beginning of the experiment and then on the 1, 7, 14, and 21 days of oral treatment. At the end of the experiment, the animals were sacrificed and analysis of liver and muscle glycogen content was carried out. Blood samples were taken from the aural vein and collected in serum separation tubes (Clot Activator & Gel, Turkey). Blood clot was removed by centrifugation at 3000 g for 10 min in a centrifuge at 4°C. The resulting supernatant was designated as a serum.

### 2.5. Study Design

The animals were randomly divided into three groups (n=9) as follows: group 1: normoglycemic, group 2: hyperglycemic control, putting immobilization, and group 3: hyperglycemic experimental, received RO seed extract (150 mg of extract per kg body weight (BW). This number of animals was chosen because they showed reliable reproducible results. Each rabbit was housed individually in a separate cage (0.90 × 0.60 × 0.40 m) with standard laboratory diet.

### 2.6. Biochemical Analysis

The biochemical analysis was performed determining the serum level of glucose, TC, HDL- cholesterol, and LDL-cholesterol. All parameters were assayed using enzymatic kit. Serum glucose level (mmol/L) was determined using glucose test kit based on the glucose oxidase method [17]. TC and TG were estimated by the method, as described [18]. HDL and LDL were measured using the method, as developed before [19]. The atherogenic index (AI) was determined by the formula, as suggested [19]. Briefly, AI = (TC – HDL) / HDL. Serum was collected and liver enzyme markers [aspartate aminotransferase (AST) and alanine aminotransferase (ALT)] were determined by kinetic UV assay using kits. Analytical tests were conducted using automatic biochemical analyzers VITROS-5.1/FS (Germany) and MINDRAY B1-120 (China).

### 2.7. Oral Glucose Tolerance Test

On the 10^th^ day of treatment an oral glucose tolerance test (OGTT) was carried out. Animals were fasted overnight before commencing of experiments. 20% glucose solution (2g/kg BW) was administrated a signal oral dose to all groups of rabbits. The blood glucose level was measured by portable glucometer (Contour TS, Bayer, Switzerland). Blood samples were collected from aural vein at 0, 30, 60, 90, and 120 min after glucose loading. Glucose clearance was evaluated by calculating the area under the curve (AUC) of the glycemic profile.

Total glycemic responses to OGTT were calculated from respective areas under the curve for glucose (AUC_glucose_) by trapezoid rule for the 120 min [20].

### 2.8. Histopathological Examination of Tissue Samples

The liver and muscles of experimental animals were harvested and followed by the histopathological examination; glycogen contents were determined by the method, as described in [21].

### 2.9. Data Processing

All values were expressed as mean ± SEM for three rabbits in each group. Data processing was done using “Statistica 6.0” software for Windows. The differences between the results of different series were considered valid if student criteria (p) was <0.05. A difference of p < 0.05 or less in the mean values was considered as statistically significant.

## 3. Results 

### 3.1. Phytochemicals in R. obtusifolius

TPC determination has shown a significant concentration of phenolics in RO extract of 327.2±0.33 *μ*g GAE/mg DW. Flavonoids and tannins contents were of 47.37±1.23 *μ*g GAE/mg DW and of 23.93±0.26 CE/mg DW, respectively. It should be noted that there is no literature data about phytochemical composition of RO extracts; however, it is well known that plants of the genus are abandoned with anthraquinones, flavonoids, terpenoids, and carotenoids [10]; detailed phytochemical analysis will be done in the following study.

### 3.2. Effect of R. obtusifolius on Fasting Glucose Levels

**Figure 1:**
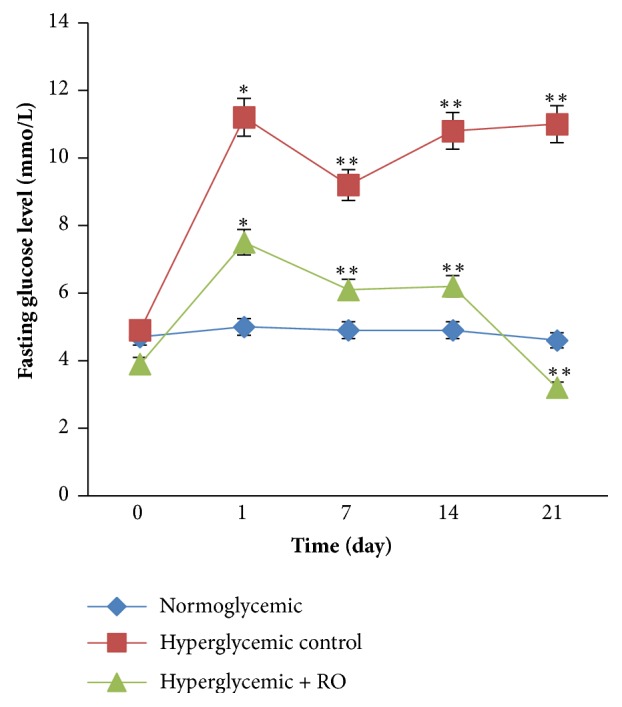
Effect of RO ethanol extract on fasting blood glucose levels in normoglycemic and hyperglycemic rabbits. Data are represented as mean ± SEM for 3 animals per group. *∗*Significantly different levels compared to the normoglycemic group (p<0.05). *∗∗* Significantly different levels compared to the hyperglycemic control group (p<0.05).

Antihyperglycemic activity of RO seeds ethanol extract was evaluated on hyperglycemic rabbit model. Fasting blood glucose levels in the hyperglycemic control (56.2%) and hyperglycemic + RO extract (48.0%) groups during the first day of immobilization (5 h) were significantly increased, compared to the normoglycemic group, p<0.05 (Figure 1). Therefore, it may be noted that disposable strong stressful pressure provokes of hyperglycemia. Treatment with RO (150 mg/kg BW) single dose for 21 days showed a significant reduction in fasting glucose to hyperglycemic rabbits (57.3%, p<0.05) compared to the 1^th^ day value.

### 3.3. Effect of R. obtusifolius on OGTT

**Figure 2:**
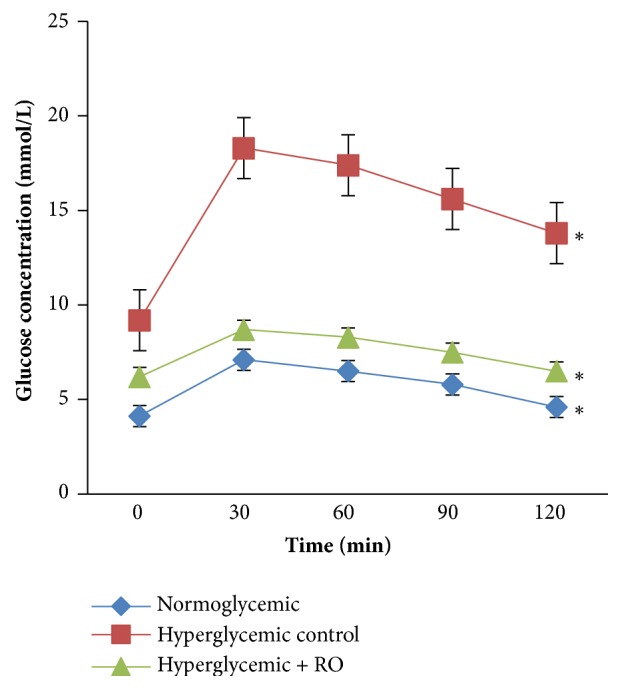
Effect of RO on OGTT in normoglycemic and hyperglycemic rabbits. Data are represented as mean ± SEM for 3 animals per group. *∗*Significantly different levels compared to the normoglycemic group (p<0.05).

It was observed that the seeds extract showed a significant effect on hyperglycemia compared to the hyperglycemic group (Figure 2). Blood glucose in all groups were increased at 30 min time point after glucose load, and then gradually decreased following hours. At 120 min blood glucose levels were significantly reduced in treated group of rabbits (25.3%) and hyperglycemic control group (14.7%) compared to the values at 30 min (see Figure 2). Therefore, glucose tolerance was significantly improved in the RO treated animal groups, compared to the hyperglycemic control group (p<0.05).

**Figure 3:**
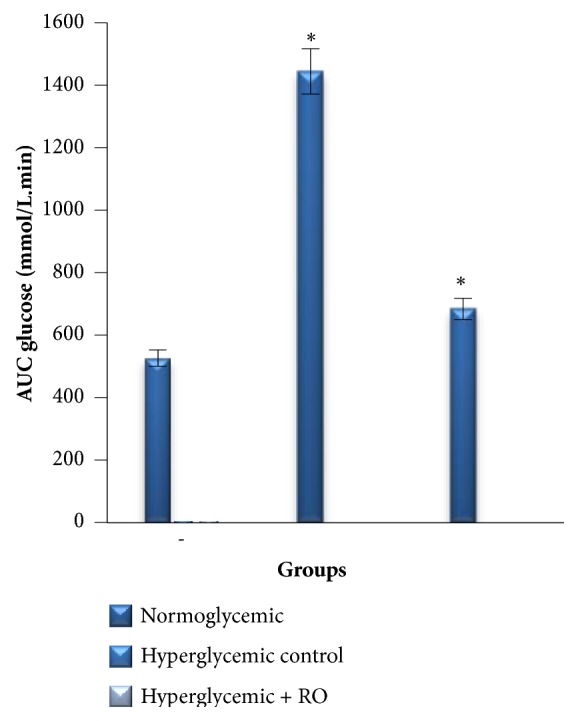
Area under curve for glucose (AUC_glucose_) from OGTT graph of normoglycemic and hyperglycemic rabbits. Data are represented as mean ± SEM for 3 animals per group. *∗*Significantly different levels compared to the normoglycemic group (p<0.001).

The area under the curve (AUC) is represented in Figure 3. The AUC_glucose_ value for glucose at 0 to 120 min was significantly increased in the untreated animals while glucose concentration was decreased to 52.6% (p<0.001) for treated group compared to the hyperglycemic control group.

### 3.4. Effect of Ethanol Extract of R. obtusifolius on Serum Lipid Profiles

The data showed that the TC and LDL-cholesterol levels in the hyperglycemic control group were significantly increased (68.8% and 61.9% respectively), TG level was increased (17.5%) compared to the normoglycemic group (Table 1). After 21 days of oral treatment, physiological levels of blood lipids parameters demonstrated significantly decreased TC and LDL-c levels (53.3% and 38.4%, respectively), and reduced TG level (17.5%) compared to the hyperglycemic control group. The HDL levels of the extract-treated hyperglycemic group did not differ significantly from the hyperglycemic animals group. As concerns, AI the hyperglycemic control group of animals demonstrated increased (88.5%), compared to the normoglycemic group.

### 3.5. Effects of R. obtusifolius on Liver Enzymes

**Figure 4:**
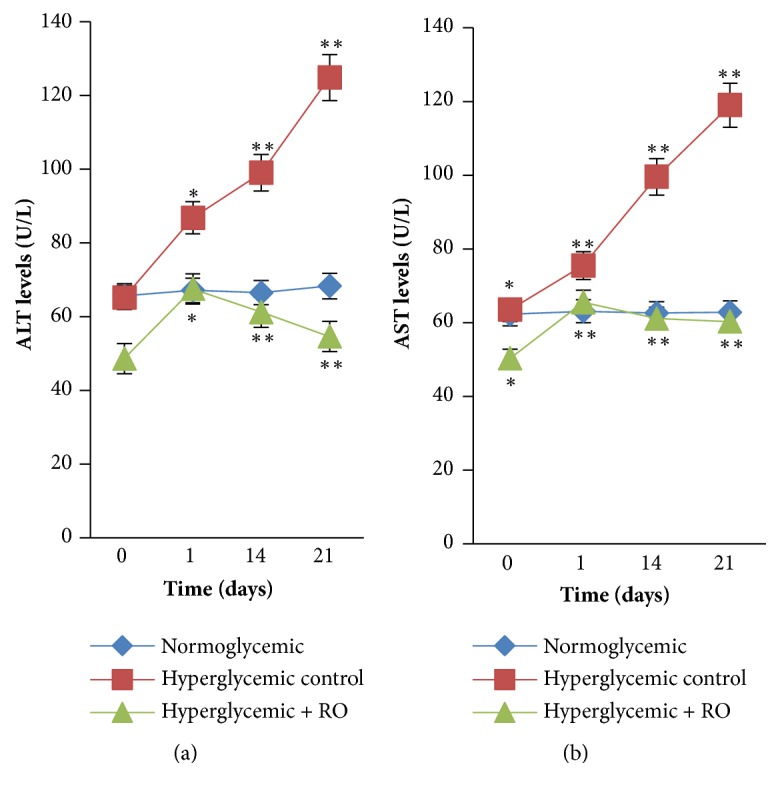
Effect of RO ethanol extract on liver enzyme (ALT, (a)) and (AST, (b)) levels. *∗*Significantly different levels compared to the normoglycemic group (p<0.05). *∗∗* Significantly different levels compared to the hyperglycemic control group (p<0.05). Data are represented as mean ± SEM for 3 animals per group.

As Figure 4 shows, liver enzymes were increased in hyperglycemic control group compared to the normoglycemic group. Serum ALT significantly increased in the hyperglycemic control group (45.3%) when compared with the normoglycemic group. Treatment with RO reduces ALT level in comparison with normoglycemic group (20%). AST level significantly increased in the hyperglycemic control group (47.2%) compared to the normoglycemic group whereas RO ethanol extract treatments reduced serum AST level in comparison with hyperglycemic control group (49.3%, p<0.05).

### 3.6. Effect of the Ethanol Extract of R. obtusifolius on Liver and Muscle Glycogen Contents

**Figure 5:**
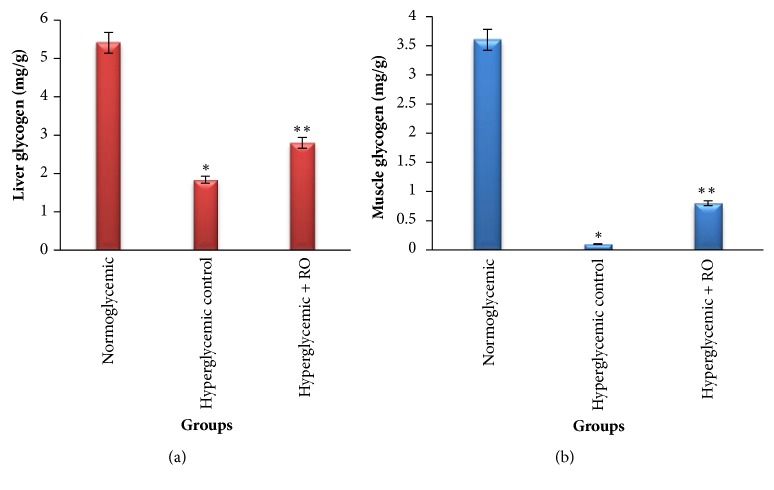
Effect of RO ethanol extract on liver (a) and muscle glycogen (b) levels. Data are represented as mean ± SEM for 3 animals per group. *∗*Significantly different levels compared to the normoglycemic group (p<0.01). *∗∗*Significantly different levels compared to the hyperglycemic control group (p<0.01).

Glycogen levels were depleted in both hyperglycemic control and hyperglycemic + RO groups (3.0-fold and 1.5-fold, respectively) compared to the normoglycemic group (p<0.01) (Figure 5). However, the hyperglycemic + RO group induced increases in the liver glycogen levels compared to the hyperglycemic control group (1.5-fold, p<0.01) (see Figure 5(a)). The hyperglycemic control and hyperglycemic + RO groups showed strong reduction (36-fold and 4.5-fold respectively, p<0.01) in muscle glycogen levels, compared to the normoglycemic group (see Figure 5(b)). The increase in liver glycogen levels may suggest that RO seeds extract stimulated insulin secretion from pancreatic *β*-cells, therefore, enhancing the impaired capacity of the liver to synthesize glycogen [22, 23].

### 3.7. Effect of R. obtusifolius on Body Weights Change

**Figure 6:**
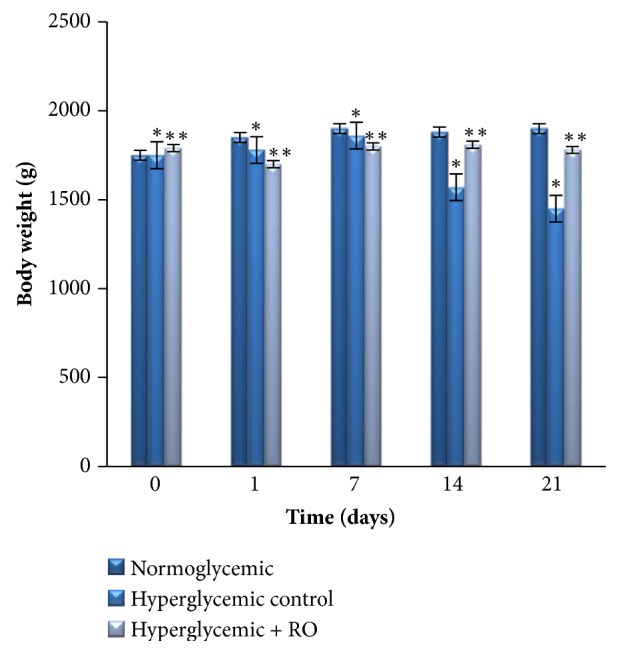
Effect of RO ethanol extract on the body weights of rabbits. Data are represented as mean ± SEM for 3 animals per group. *∗*Significantly different levels compared to the normoglycemic group (p<0.01). *∗∗* Significantly different levels compared to the hyperglycemic control group (p<0.01).

The effect of ethanol extract of RO seeds on body weight in experimental rabbits was summarized in Figure 6. During the 21 days of study, animals in normoglycemic group continued to gain weight by 7.89%, whereas hyperglycemic control group continuously loss weight (21.62%) compared to the initial day. There was no significant decrease in the body weight of extract-treated animals when compared with the starting day.

## 4. Discussion

Diabetes is a disease that affects many people in the 21^st^ century. The number of people with diabetes has been growing and causing increasing concerns in medical sphere and the public [1]. Type 2 diabetes mellitus (T2D) is more prevalent form and it is expected to reach pandemic levels. This issue is spread in Armenia consequently different factors like aging, stress, sedentary lifestyle, and unhealthy food. T2D is a chronic disease characterized by insulin resistance which leads to hyperglycemia.

Hyperlipidemia is one of the major factors linked with hyperglycemia due to insulin deficiency during diabetes and correlated with carbohydrate metabolism. Insulin resistance and lack of insulin secretion due to pancreatic *β*-cell failure are among the leading causes of type 2 diabetes [24, 25].

In this study, hyperglycemia in rabbits was induced by immobilization stress. The latter leads to disorder of the endocrine system, especially increase the blood glucose levels and lipid metabolism consequently to the increase of TG, LDL-cholesterol. and to the decrease HDL-cholesterol levels. Probably prolonged immobilization stress increases the risk of cardiovascular disease due to impaired endothelial function leads to development of atherosclerosis, which has an endothelial dysfunction at early stages [7, 8].

Previously, it was observed that aqueous extract of hydroponic* Stevia rebaudiana* that possess antihyperglycemic and antihyperlipidemic activities has hepatoprotective effect in hyperglycemia induced by immobilization stress in rabbits [26]. Wild species of* Rumex* are widespread in the Armenian flora and are used for the prevention and treatment of various diseases; however antihyperglycemic properties of this plant have not been sufficiently studied.

In traditional medicine numerous* Rumex *species are used, as anti-inflammatory agents. The decoction of the seeds of* R. obtusifolius* is used for the treatment of coughs of all types, colds and bronchitis [10], and renal and urogenital disorders [27], and also to control mild forms of diabetes [27, 28]. Many findings revealed that leaves of* Rumex *species are most frequently utilized plant parts as foods, while roots and seeds are applied preferably for the treatment of different diseases [10]. Roots are mainly used for the treatment of constipation, seeds in case of diarrhea and leaves for the therapy of skin disorders [10]. Pharmacological investigations have shown that the crude extracts and isolated compounds from* Rumex *species possess different kinds of biological activities, especially antioxidant [29–31], antitumor [32], anti-inflammatory, antiulcer, and antimicrobial effects [11].

Some* Rumex *species are rich in flavonoids and other phenolic compounds which have shown antioxidant properties [11, 29, 31]. The highest amount of total phenolic compounds was found in the ethanol extract of the* Rumex crispus* seeds [31].* R. obtusifolius* revealed antibacterial activity and also it could verify its traditional use for the treatment of several skin diseases [13, 31].

Due to the high tannin content the roots of some* Rumex *species may have considerable carcinogenic potential [10]. Methanol extract of RO leaves showed the highest antioxidant activity due to phenolic content [11, 13]. This is likely to our data about high phenolic content obtained (see Results). Lone et al. [33] reported that the ethanol extract of* Rumex patientia* roots has protective effect against the oxidative damage of lipids and DNA and restores AST, ALT, ALP, and bilirubin levels. The seeds of* R. patientia *have a hypoglycemic effect and improve serum lipid profile in regard to HDL- and LDL-cholesterol levels [34].

Our findings showed that RO extract has a high concentration of phenolic compounds, flavonoids, and tannins (see the Results). These substances might be responsible for hypoglycemic effects of RO. Among the phenolic compounds, the flavonoids act as insulin secretagogues that may improve glucose uptake in peripheral tissues. Also the flavonoids may regulate the activity of rate-limiting enzymes involved in carbohydrate metabolism pathways [35]. Tannins are also phenolic compounds and commonly used as healing agents [16]. The new detections may increase the therapeutic importance of* Rumex *species and encourage their future use in modern medicine.

In the current study we found a positive effect of the ethanol extract of RO seeds on blood glucose levels, lipid parameters, liver function enzymes (ALT, AST), glycogen content, and body weights in hyperglycemia induced by immobilization stress in rabbits for 21 days. Our results showed that the rabbits in the hyperglycemic control group demonstrated increased blood glucose and lipids levels and loss of body weight compared to the normoglycemic group. It was noted that strong stressful pressure provokes hyperglycemia. However, the oral treatment with the RO extract at the 21^st^ day demonstrated reduction in fasting glucose level (see Figure 2). This reduction may be due to the inhibition of GP-*α* and *α*-glucosidase activity to enhance glycogen synthesis and slow down digestion of carbohydrates, thereby improve regulation of glucose in diabetic condition [36]. The blood glucose lowering effect of RO seeds extract could be attributed to the presence of flavonoids and phenolic compounds that have been associated with hypoglycemic activity [10]. Our study demonstrated that ethanol extract of RO seeds, perhaps, stimulated insulin secretion, and peripheral glucose utilization, improving the hyperglycemic condition.

It was observed that treatment with the seed extract of RO increased glucose tolerance compared to the untreated group of animal (see Figure 2). OGTT was used to identify the altered carbohydrate metabolism during post glucose administration. The ability of extract to lower the blood glucose level in oral glucose tolerance test suggests that rabbits treated with extract had better glucose utilization capacity. Treatment with RO extract of hyperglycemic rabbits significantly reduced blood glucose levels and increased glucose tolerance during OGTT.

Abnormal lipid metabolism leading to increasing of several serum parameters, including increase in TC, TG, and LDL-cholesterol levels, and reducing in HDL-cholesterol level are hyperglycemia indicators. They are included in increased risk of clinical diseases [4, 26, 37, 38]. Excess LDL-cholesterol can be deposited in blood vessel walls, directly inducing the formation of atherosclerosis [38]. HDL-cholesterol has protective effects because it transfers cholesterol from peripheral tissues to the liver through the reverse cholesterol transport “pathway for catabolism” [5, 39]. TG levels play key role in the regulation of lipoprotein interactions in maintaining normal lipid metabolism and have also been proposed as major determinants of cholesterol esterification [5, 26, 38, 39].

In the current study a reduction in the TG, TC, and LDL-cholesterol after treatment with RO extract it was observed (see Table 1). Hypolipidemic activity of the RO extract may be mediated by reducing intestinal cholesterol absorption and increasing reverse cholesterol transport. The mechanisms of hypolipidemic effects of RO extract are not known but could possibly be due to the biological active compounds present in RO. Therefore, it was also found that RO seed extract improved the serum lipid profile, which eventually alleviated diabetes complications. The detailed analysis of the extract is important and further research will be devoted to not only to the study of phenolic profile of this extract, but also of other secondary metabolites.

Insulin is stored in large dense core vesicles in the pancreatic *β*-cells and secreted by exocytosis in response to different hormonal modulators [23, 36, 40]. The reduction of liver glycogen due to lack of insulin or insulin resistance is often linked with enhanced activity of glycogen phosphorylase to improve glycolysis, eventually resulted in hyperglycemia [33, 41, 42]. However, it is known that insulin stimulates glycogen synthase and inhibits glycogenolysis in the liver. Insulin deficiency results in an inactivation of glycogen synthase and enhances glycogenolysis, thereby decreasing liver glycogen in hyperglycemic animals [41, 42].

Our study has shown that as a result of immobilization, liver and muscle glycogen content were reduced which could be linked to an inactivation of glycogen synthase. However, our results indicated that administration of RO increased liver glycogen level (see Figure 5(a)), compared to the untreated hyperglycemic rabbits. It may suggest that administration of RO stimulated insulin secretion from pancreatic *β*-cells, thereby increased liver glycogen synthesis [2, 23, 42]. There were no significant differences between the muscle glycogen levels of the hyperglycemic control and treated group (see Figure 5(b)). In further research hormonal metabolism, particularly, insulin level in serum will be studied and histopathological examination of the pancreatic islets will be done.

Liver enzymes (ALT, AST) were investigated in this study as marker for hepatic diseases and hepatic damage. They are released into the blood stream after cell membrane damage [42]. In the present study, the activity of these enzymes in serum of hyperglycemic control group of rabbits was higher compared to the hyperglycemic + RO group, which indicates that the immobilization stress had severe liver cell damage. After treatment the activities of ALT and AST significantly decreased (see Figure 4). Therefore, RO extract demonstrated an attenuation effect on hepatic damage.

The body weight lowering is often associated with hyperglycemic conditions, as a result of insulin deficiency which produces degeneration of structural proteins and resulted in muscle wasting [24, 39]. However, it was observed that, during the experimental period, body weight of RO treated rabbits did not change compared to initial date (see Figure 6). It is suggested that the RO seeds extract could be protective against the degradation of structural proteins.

## 5. Conclusions

The present study has shown the efficacy and safety of the ethanol extract of* R. obtusifolius* seeds in the treatment of hyperglycemia. The extract revealed hypoglycemic activity, improved lipid profile and body weight, corrected liver enzymes activities, and restored liver and muscle glycogen levels in hyperglycemia induced by immobilization stress in rabbits.

Further investigations on the chemical compounds responsible for these effects should be performed to clarify the mechanisms of action.

## Figures and Tables

**Table 1:** The effect of RO ethanol extract on lipid parameters in rabbits.

Parameters (mmol/L)	Experimental groups		
	Normoglycemic	Hyperglycemic control	Hyperglycemic + RO
TC	1.4 ± 0.08	4.5 ± 0.16 *∗*	2.1 ± 0.09*∗*
TG	0.85 ± 0.12	1.03 ± 0.4 *∗*	0.85 ± 0.05 *∗*
HDL	0.78 ± 0.04	1.02 ± 0.15*∗*	1.04 ± 0.08*∗*
LDL	1.0 ± 0.15	2.63 ± 0.09*∗*	1,.62 ± 0.08*∗*
VLDL	0.17 ± 0.03	0.46 ± 0.07*∗*	0.38 ± 0.02*∗*
AI	0.4 ± 0.05	3.5 ± 0.15*∗*	1.1 ± 0.05*∗*

*∗* Significantly different from normoglycemic group (p<0.05). Data are represented as mean ± SEM for 3 animals per group.

## Data Availability

All data analyzed during this study are included in this article. The data supporting the conclusions of this article are included within this article.

## Abbreviations

AI: Atherogenic index
ALT: Alanine aminotransferase
AST: Aspartate aminotransferase
AUC: Area under the curve
DM: Diabetes mellitus
HDL-c: High-density lipoprotein cholesterol
LDL-c: Low-density lipoprotein cholesterol
RO: * Rumex obtusifolius*
TC: Total cholesterol
TG: Triglycerides
TPC: Total phenolic content
VLDL-c: Very low-density lipoprotein cholesterol.
